# The Clinical Effectiveness of Patient Initiated Clinics for Patients with Chronic or Recurrent Conditions Managed in Secondary Care: A Systematic Review

**DOI:** 10.1371/journal.pone.0074774

**Published:** 2013-10-07

**Authors:** Rebecca Whear, Abdul-Kareem Abdul-Rahman, Kate Boddy, Jo Thompson-Coon, Mark Perry, Ken Stein

**Affiliations:** 1 Peninsula Collaboration for Leadership in Applied Health Research and Care, Institute of Health Research, University of Exeter, Exeter, Devon, United Kingdom; 2 Rheumatlogy Department, Plymouth Healthcare National Health Service Trust, Plymouth, Devon, United Kingdom; University of New South Wales, Australia

## Abstract

**Background:**

Missed or inappropriate hospital appointments cost the UK National Health Service millions of pounds each year and delay treatment for other patients. Innovative methods of appointment scheduling that are more flexible to patient needs, may improve service quality and preserve resources.

**Methods:**

A systematic review of the evidence for the clinical effectiveness of patient initiated clinics in managing long term care for people with chronic or recurrent conditions in secondary care. Seven databases were searched including MEDLINE, Embase and PsycINFO (using the OVID interface), the Cochrane Library of Systematic Reviews and CENTRAL, Science Citation Index Expanded, Social Sciences Citation Index, and Conference Proceedings Citation Index (via the Web of Science interface) from inception to June 2013. Studies comparing patient initiated clinics with traditional consultant-led clinics in secondary care for people with long term chronic or recurrent diseases were included. Included studies had to provide data on clinical or resource use outcomes. Data were extracted and checked by two reviewers using a piloted, standardised data extraction form.

**Results:**

Eight studies (n = 1927 individuals) were included. All were conducted in the UK. There were few significant differences in clinical outcomes between the intervention and control groups. In some instances, using the patient initiated clinics model was associated with savings in time and resource use. The risk of harm from using the patient initiated clinic model of organising outpatient care is low. Studies with longer follow-up periods are needed to assess the long term costs and the ongoing risk of potential harms.

**Conclusions:**

The UK policy context is ripe for evidence-based, patient-centred services to be implemented, especially where the use of health care resources can be optimised without reducing the quality of care. Implementation of patient initiated clinics should remain cautious, with importance placed on ongoing evaluation of long term outcomes and costs.

## Introduction

Around 17.5 million people in the UK have a chronic condition [1]. Usually these people attend regular hospital appointments initiated by a physician (usually every six, nine or 12 months). The appointments commonly occur at a time when a person is feeling relatively well with little action taken as result. Conversely, when symptoms recur or worsen it may be difficult to obtain urgent appointments [2] as outpatient capacity is committed to routine follow-up.

Missed hospital appointments are reported to cost the UK National Health Service millions of pounds every year [3]. Traditionally many lifelong secondary care appointments for people with chronic diseases are arranged weeks or months in advance, clinician driven, and often happen at times when the patient is well. Alternative methods of appointment scheduling with improved flexibility have been developed. Some examples of this are the Choose and Book service implemented in 2004 [4] and the Expert Patient Programme courses implemented in 2006 [1]. The Expert Patient Programme aimed to help people with long term conditions develop skills and confidence to self-manage their condition and make more effective use of healthcare services. Other strategies commonly used to improve appointment attendance include: over-booking, fines for missed appointments and appointment reminder systems; these strategies are less responsive to patient need.

In 2002 the World Health Organisation published a report which highlighted the need for a model of care that more readily meets the needs of people with chronic conditions [5]. The authors of the report suggested that innovations that build on evidence-based decision-making, have a population and quality focus and are flexible to the needs and demands of the patient population should do well in improving the management of chronic conditions for the healthcare system and the people that use it.

In contrast to a traditional appointment system, a patient-initiated clinic (PIC) system aims to be responsive to patient need. Routine appointments are not regularly scheduled by the physician. An example of how this might work in practice is, if a patient is experiencing an exacerbation of their condition, they can phone an advice line manned by a specialist nurse, and where necessary a consultant appointment is arranged as soon as possible.

Several studies have assessed the effectiveness of the PIC system (otherwise known as open or advanced access) in primary care [6]–[8]. The results suggest that this method of scheduling outpatient appointments results in improvements in satisfaction with, and a reduced cost of, care delivery. With the increasing focus on NHS efficiencies highlighted by the Government's Health and Social Care Bill [9], determining the benefits and harms of alternative methods of appointment scheduling in the secondary care setting is crucial to understanding their worth for both the health system and the people who use it.

The objective of this study was to systematically review the evidence for the PIC system in secondary care for patients with chronic or recurrent conditions. In particular we were interested in whether these clinics can effectively manage conditions without causing clinical harm to patients and whether resource use can be reduced in comparison to usual care.

## Methods

The systematic review was conducted according to the principles published by the NHS Centre for Reviews and Dissemination [10]. A protocol was developed in consultation with experts and is available on the PenCLAHRC website http://clahrc-peninsula.nihr.ac.uk/systematic-review-of-patient-initiated-clinics.php.

### Literature search and eligibility criteria

**Figure 1:**
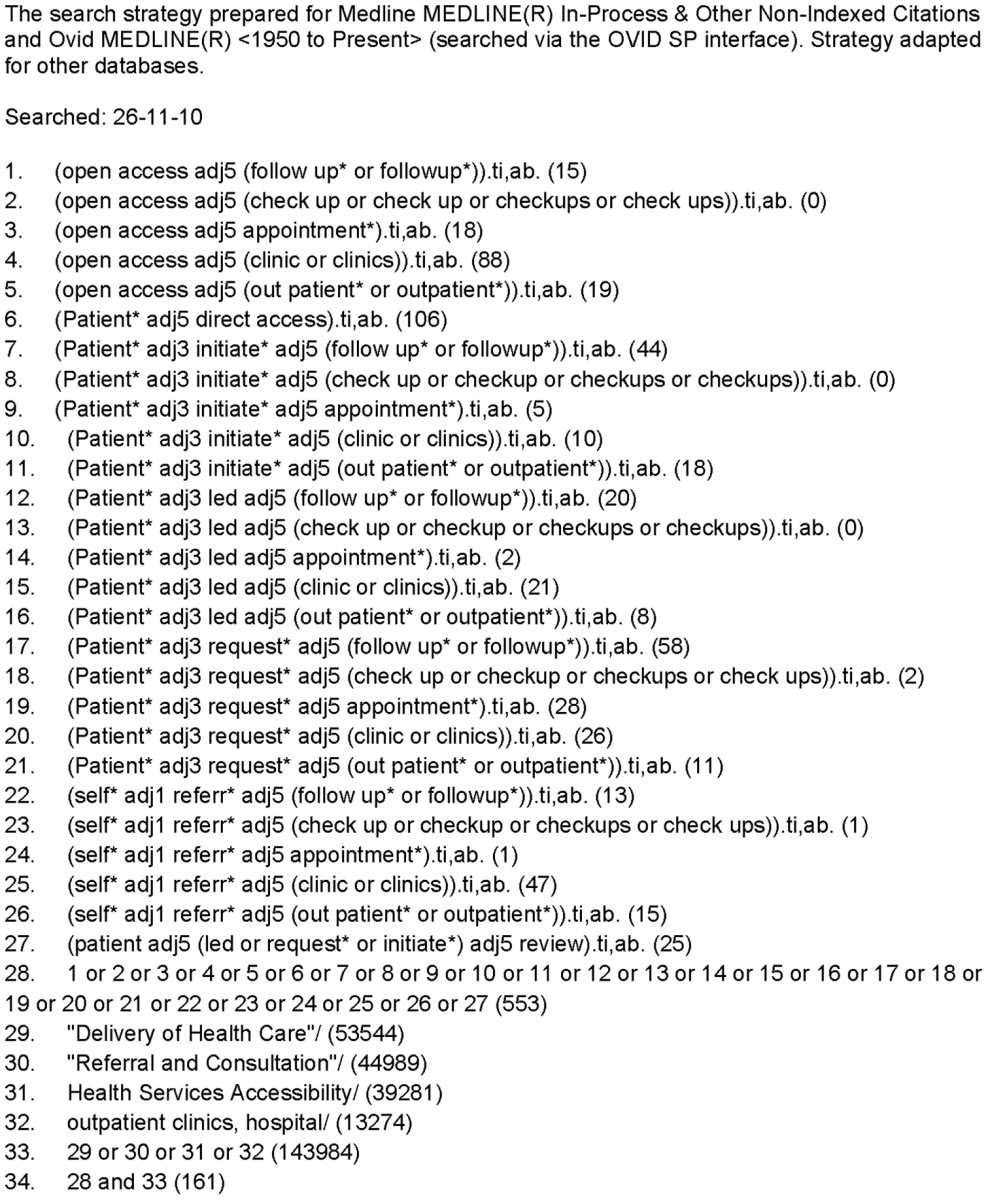
Master search strategy.

An information specialist (IS) developed the search strategy in collaboration with clinical consultants and other IS experts to ensure all appropriate terms were captured (KB). No methods filter was applied to the search strategy. The search was conducted in the following databases: MEDLINE, Embase and PsycINFO (using the OVID interface), the Cochrane Library of Systematic Reviews and CENTRAL, Science Citation Index Expanded, Social Sciences Citation Index, Conference Proceedings Citation Index (via the Web of Science interface) from inception to December 2010 (see Figure 1 for the full strategy). Update searches were conducted in October 2011 and June 2013. We also checked the references of included studies, hand searched the BMJ from 1990 to October 2011 for additional studies, searched for ongoing research studies and contacted authors and other experts to identify relevant literature. All searches were recorded and Endnote X4 was used to manage the references (KB).

Studies were included if they reported a comparison of the effectiveness of PICs (the intervention) against routine, clinician-led, follow-up procedures in secondary care for people with chronic or recurrent conditions. We defined chronic or recurrent conditions as those diseases that are of a long duration and have fluctuating symptoms, although no ‘condition’ filter was used in the search strategy. Included studies had to report at least one relevant outcome such as health status, frequency of visits, cost to patient or NHS, or other measures of disease control. Studies where there was insufficient information to allow appraisal of study quality were excluded as were studies set in primary care, those that dealt with diagnosis (as opposed to management) of conditions, and those that included short term acute conditions. No date or language restrictions were applied.

Titles and abstracts were independently screened by two reviewers (JTC, AA or RW) using the inclusion and exclusion criteria. Full texts were retrieved for articles that required more in depth application of the inclusion and exclusion criteria. All full texts were independently reviewed by two reviewers and discrepancies were resolved by a third (JTC) and fourth (KS) reviewer where necessary.

### Data collection

Data extraction was conducted by two reviewers (RW and AA) and checked by a third reviewer (JTC) using a standardised data extraction form (Table S1). The data extracted included information on the quality of the study (based on the guidelines from the Centre for Reviews and Dissemination) and information on the participants, intervention and control descriptions, outcomes and outcome measures as well as the results. Studies were not excluded on the basis of quality.

### Data synthesis

Narrative synthesis was used to summarise and discuss the results of the included studies following the principles described in Economic and Social Research Council guidelines [11] and the Centre for Reviews and Dissemination guidelines [10]. Meta-analysis was inappropriate due to the clear heterogeneity in participants, conditions and outcomes across studies.

## Results

**Figure 2:**
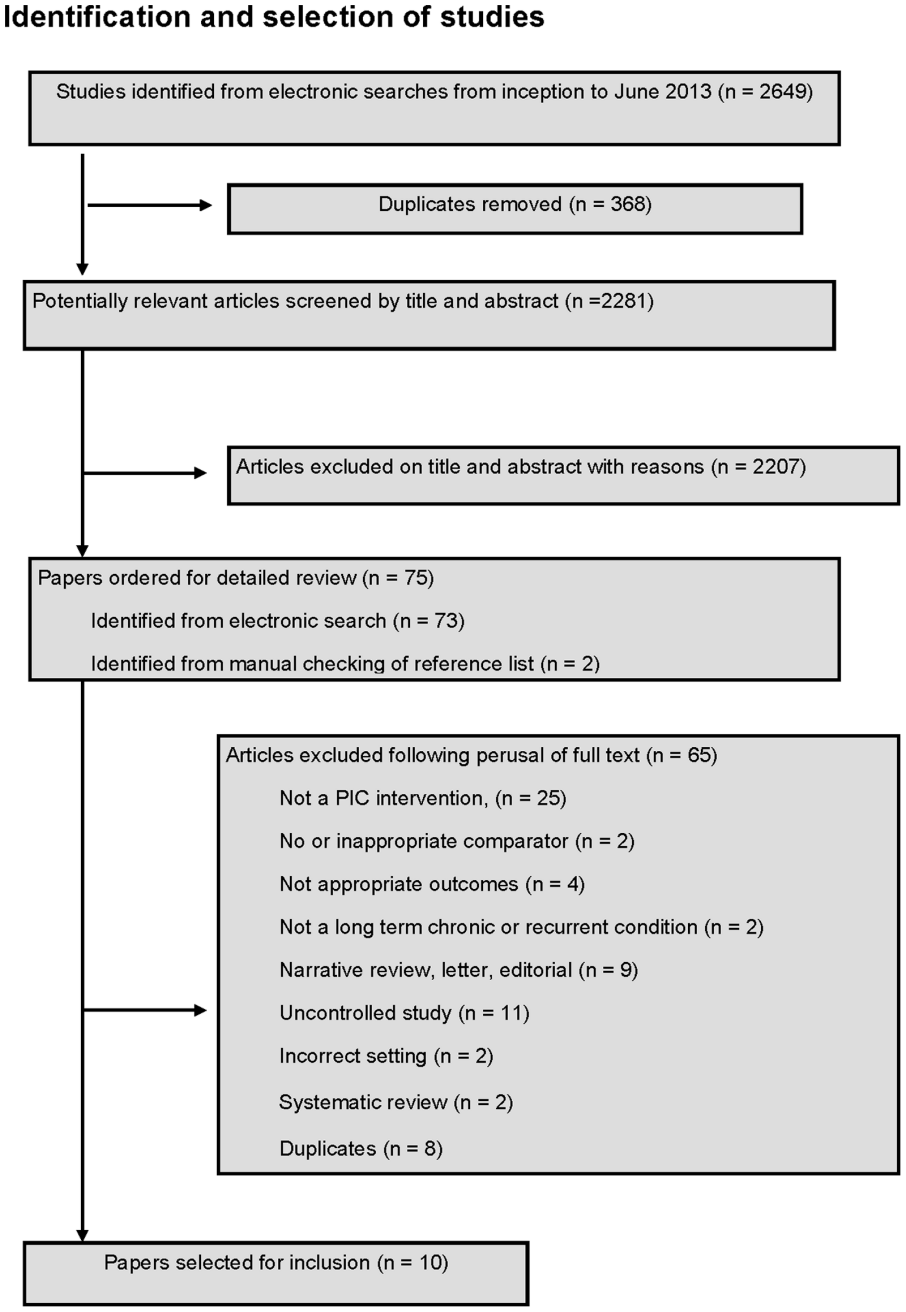
Identification and selection of studies.

The identification and selection of studies is summarised in Figure 2. Eight studies met the inclusion criteria [12]–[21], six were identified by electronic searches and two were identified by searching citations in identified studies [13], [22]. Seven included studies were randomised controlled trials (RCT) and one was a retrospective audit [18]. One study was reported in three articles at different stages of follow up [19]–[21]. In total 2,642 articles were excluded (including 368 duplicates). Reasons for exclusion after retrieval of the full text articles are presented in Figure 2.

**Figure 3:**
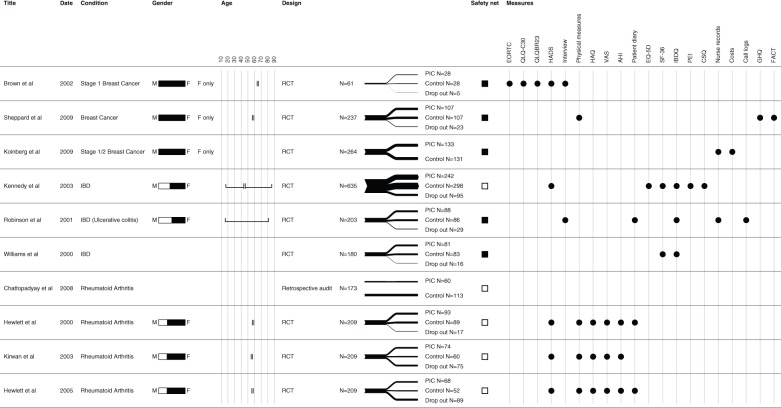
Characteristics of included studies.

The included studies reported the clinical effectiveness of a PIC system in three disease areas – breast cancer (BC) [12], [13], [22], inflammatory bowel disease (IBD) [15]–[17] and rheumatoid arthritis (RA) [18]–[21]. All of these studies were conducted in the UK. A total of 1,927 patients over the age of 16 years were included (562 with breast cancer, 1,083 with IBD, and 382 with RA). Participants were followed up for between 12 and 72 months. Figure 3 details the characteristics of the included studies.

The structure of PICs was broadly similar across the studies with the main access point being a telephone number, through which patients could request clinical advice and, if necessary, arrange an appointment to see a clinician. In four studies [12], [13], [15], [16] patients in the intervention group were also given written guidance to help them self-manage their condition and to make them aware of what symptoms or events should initiate the need for a consultation. The characteristics of the PIC system in each study are presented in Table S2.

**Figure 4:**
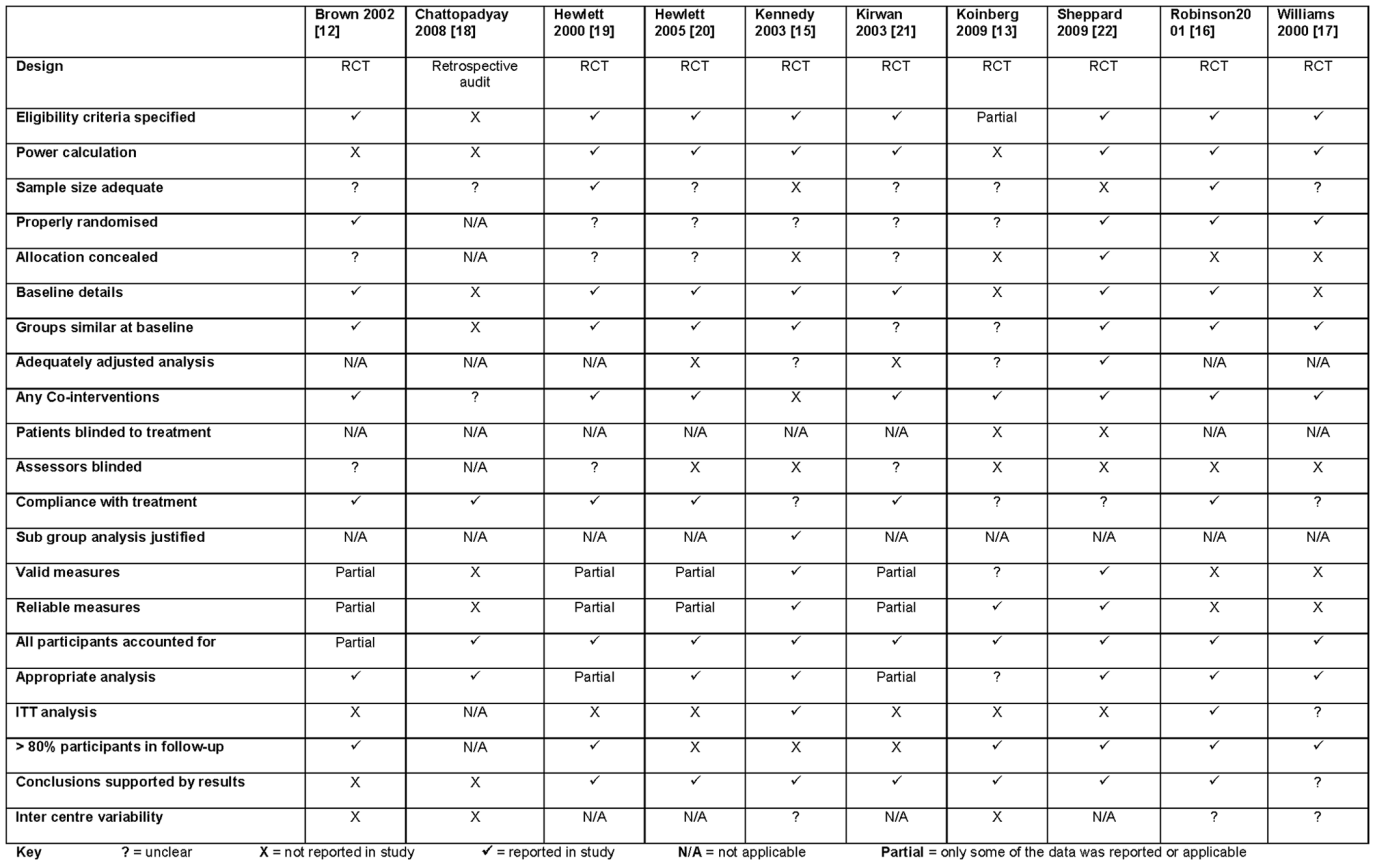
Quality of studies in included.

The quality of included studies varied (Figure 4). Despite the majority being RCTs, the quality of reporting was inconsistent and important information was lacking. For example, very few studies reported the method of randomisation; in all studies it was unclear whether the personnel measuring outcomes were blinded to the participants' allocation; information on the reliability and variability of outcome measures was not provided; very few studies reported an intention-to-treat analysis and only half reported having more than 80% of the participants remaining at follow-up. The lack of information limits the interpretation and extrapolation of the results. However details of power calculations to understand the sample size, baseline characteristics of all the participants; eligibility criteria of participants involved; and reporting of data ensuring all participants had been accounted for were well reported. The methods used and reported by Sheppard and colleagues [22], Robinson and colleagues [16], Kennedy and colleagues [15] and Hewlett and colleagues [19], [20] suggested lower potential for bias in their results.

**Figure 5:**
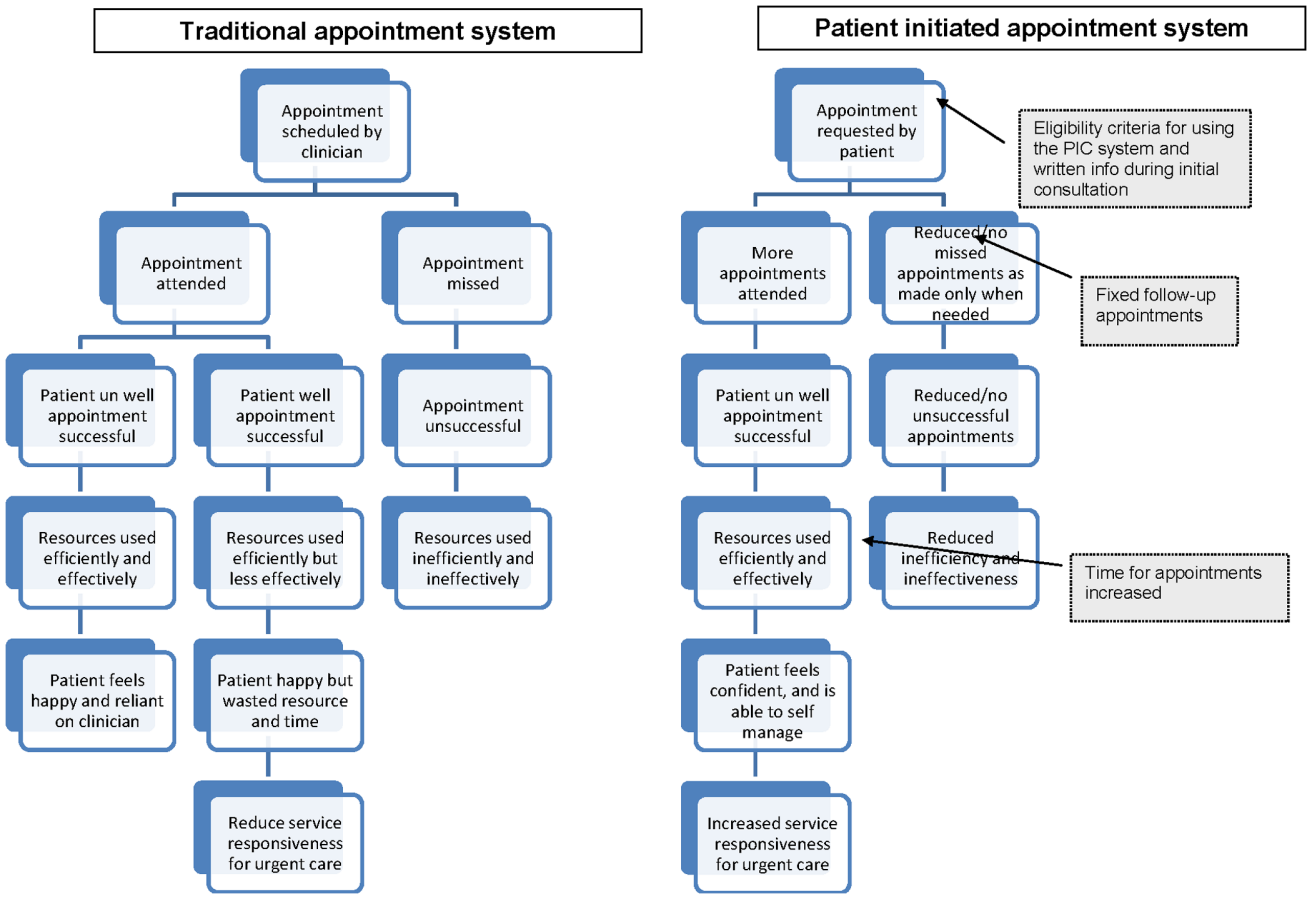
Logic model of traditional and Patient Initiated Clinic appointment systems.

**Figure 6:**
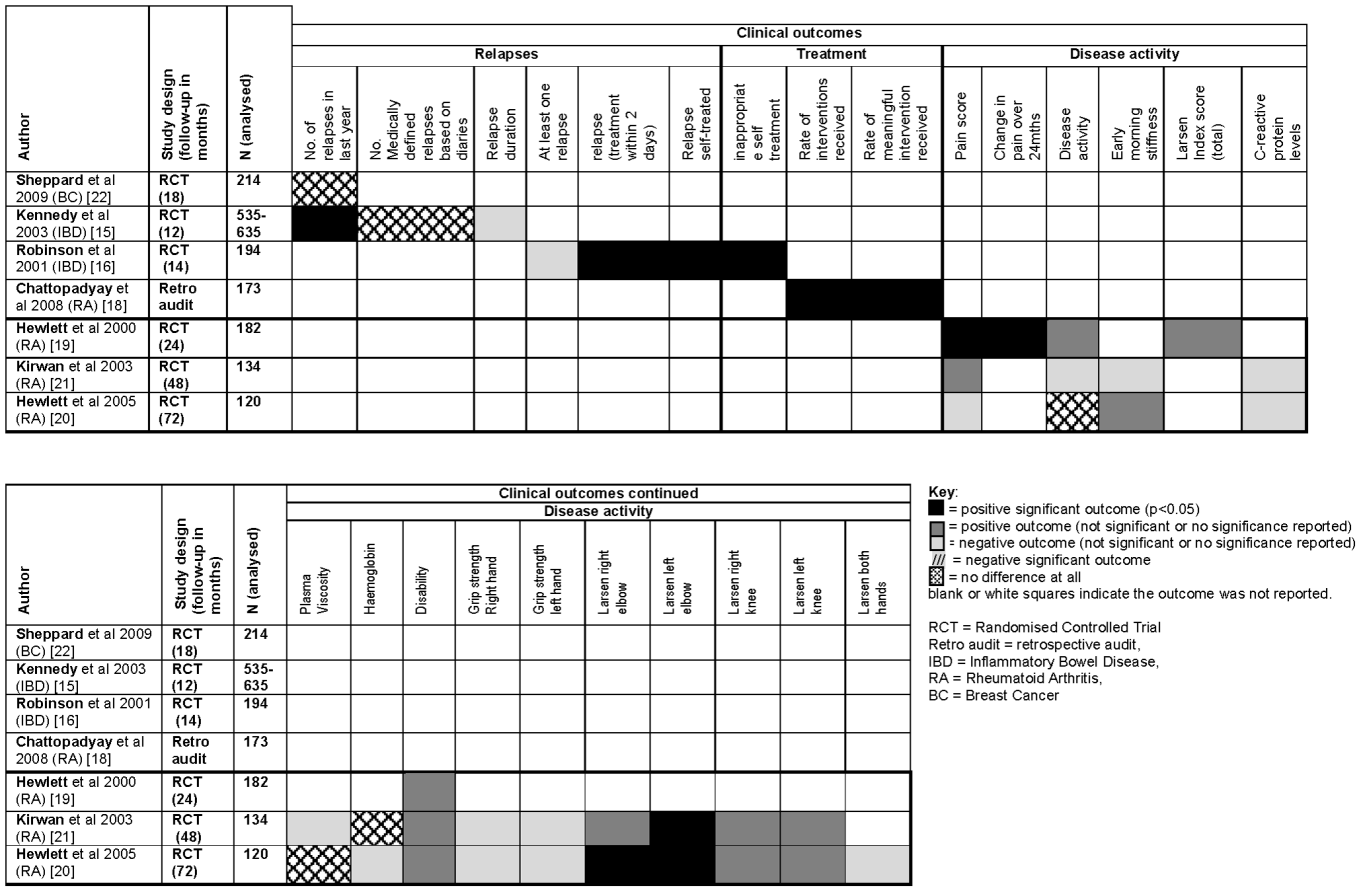
Clinical outcomes chart.

**Figure 7:**
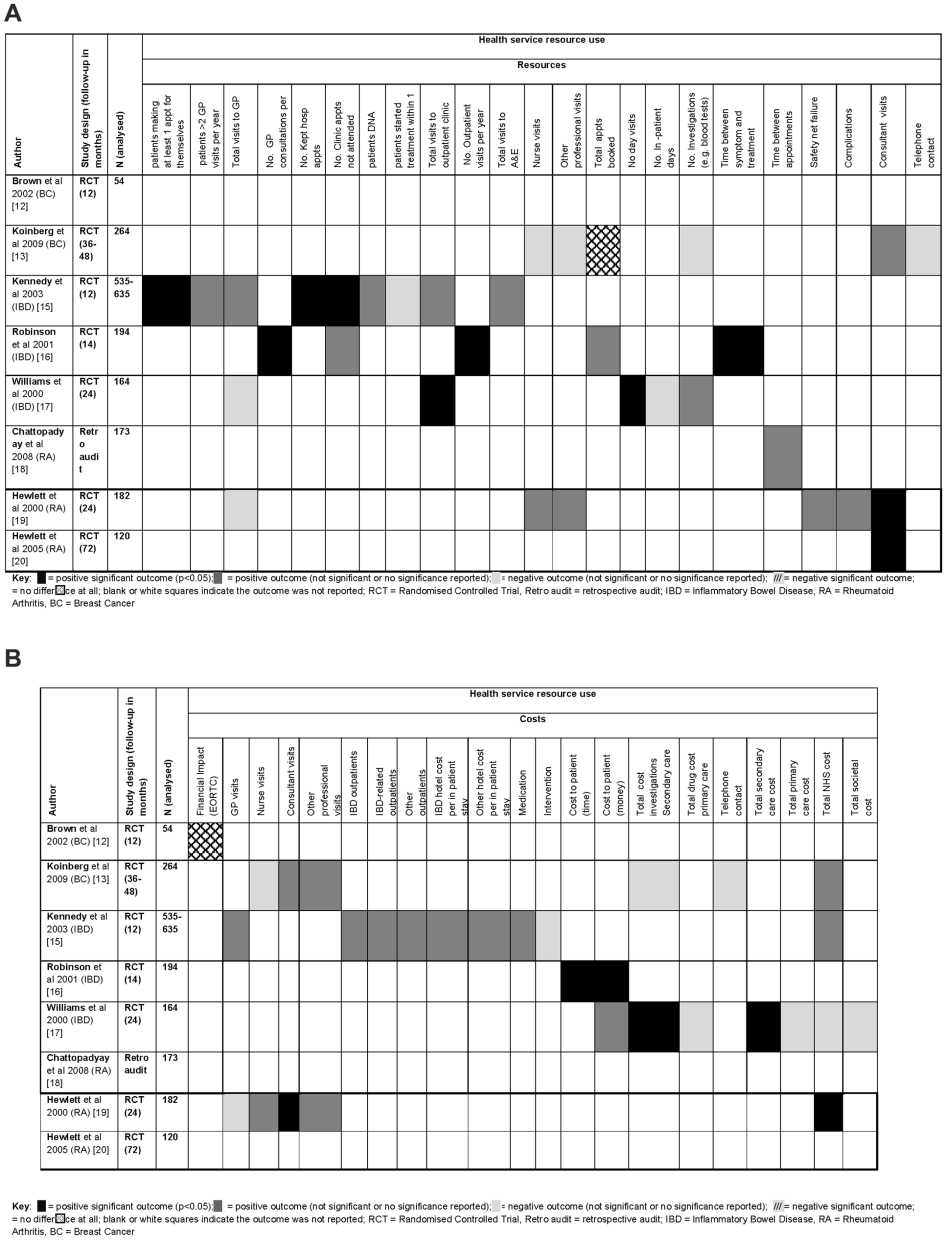
a Health service resource use and cost chart. B. Health service resource use and cost chart.

A proposed logic model for the theory behind both the traditional and PIC appointment systems is illustrated in Figure 5. The results of individual studies are presented in Figures 6 and 7. Each figure lists the studies that reported on the appropriate outcome data across disease area. Studies that did not report on those outcomes are not included in the tables. Results as published in the original articles can be seen in Table S3.

### Clinical outcomes

A large number of clinical outcomes were reported across studies. However, there were few common outcomes and direct comparison between studies is limited.

#### Relapse

The results from Sheppard and colleagues [22] suggest that for BC patients there was no difference in the relapse rate reported between the two groups over their 18 month study (both were low at 4% recurrence). Results from one IBD study [15] suggested that at 12 months, patients in the intervention group (PIC) had a significantly reduced mean relapse rate (m = 1.8±2.2) compared with the control group (m = 2.2±2.5; 95% CI -0.63 to −0.09). In another IBD study (16), the duration of the relapse was longer (though not significantly so) for the patients in the intervention group compared to the control group. Relapse was not reported in the RA studies.

#### Medical intervention

Results from one of the studies in RA [18] implied that patients in the intervention (PIC) group received significantly more interventions in their care as a proportion of total clinic appointments than those in the control group (96.7% vs 52.2% respectively, p<0.00001). The intervention group were also more likely than the control group to receive a *meaningful* intervention, for example, a change of medication or further investigations (66.7% vs 30.1%, p<0.00001).

#### Clinical outcomes over time (RA only)

For the remaining RA studies [19]–[21] that reported on clinical outcomes it is possible to follow the same groups of participants throughout their six year follow-up, during which time no clear and consistent pattern favouring either approach to follow up was shown. Hewlett and colleagues [19] reported that at 24 months the intervention (PIC) group had statistically significant and clinically improved mean pain scores (on a 10 point scale) than the control group (m = 3.9; 95%CI 3.2 to 4.4 vs m = 4.8; 95%CI 4.2 to 5.4 respectively; p<0.05). However, by 48 months Kirwan and colleagues' [21] results indicated that the overall change in pain scores from 0–48 months was only marginally better for the intervention than the control group. The decline in pain continued in both groups up to 72 months with Hewlett and colleagues [20] reporting worse (but non-significant) change in pain scores for the intervention group (median change  = 1.25, range −0.40 to 3.25 vs 1.1, −1.00 to 3.60 respectively, p = 0.91).

Disease activity fluctuated over time, with results across the three studies [19]–[21] indicating that at 24 months, disease activity was better in the intervention group than in the control group [19], and at 48 months, disease activity was worse in the intervention group [21]. By 72 months, although both groups outcomes were worse, there was almost no difference in their disease activity [20]. These results showed no clinically significant impacts for the patients in either group. Change in disability scores held a more stable pattern, the results were better for the intervention group than the control group, although over time they both became worse. Results for other bodily functions such as hand grip strength, elbow mobility and knee mobility also presented fairly consistent results across time with the intervention group generally doing better than the control group (details can be seen in Figure 6).

### Health service resource use

#### Breast Cancer

One study on BC [13] reported a reduction in the number of consultant visits for the intervention group but an increase in the number of nurse visits and medical investigations, though these were not statistically significant. There were also increased numbers of investigations and no difference in the number of booked appointments between the intervention and control groups [13].

#### Inflammatory Bowel Disease

For patients with IBD, in the intervention group the number of patients making appointments for themselves (43% vs 22%, 95%CI 1.63, 4.46) was significantly lower than those recorded in the control group [15]. The number of clinic appointments not kept, visits to outpatient clinics, day visits and visits to the GP were reduced in the intervention group. Robinson and colleagues [16] reported that the time between symptom flares and treatment was significantly reduced for patients in the intervention group in comparison to the control group (m = 14.8 hrs vs m = 49.6hrs respectively; p<0.0001). In a third study, there was a reduction in the mean number of visits to accident and emergency wards (m = 0.03 vs m = 0.05) [15], a reduction in the number of missed appointments (n = 1 vs n = 47) [16], and a reduced number of investigations e.g. blood tests in the intervention group (p = 0.09–0.81) [17].

Williams and colleagues [17] reported that on average patients in the intervention group spent more days as inpatients than the control group (m = 0.83±3.53 vs m = 0.43±1.74 respectively; p = 0.71).

#### Rheumatoid Arthritis

Hewlett and colleagues [19], [20] reported a significant reduction in the number of consultant visits in the intervention group at 24 and 72 months in comparison to the control group (PIC median 8 (5–13) vs 13 (11–17) appointments; p<0.0001). There was also a greater time between appointments (fewer appointments being made) [18], a reduction in safety net failure and fewer complications in the intervention group [19] (Figures 7a and b).

### Health service costs

The overall cost to the NHS per patient was reported to be less in the intervention group than the control group in the four studies that measured this outcome [13], [15], [17], [19]. The annual NHS cost difference between the intervention and control groups was estimated to be an average of £89 per patient/per year (see Table 1).

**Table 1:** NHS summary costs.

Study	Control (£)	PIC (£)	Difference	Annualised cost difference (£)
Koinberg (2009) 48mths *NHS* – *BC*	1869	1266	603	−150.75
Hewlett (2000) 12mths *NHS – RA*	313	208	105*	−105
Kennedy (2003) 12mths *NHS – IBD*	1070	922	148	−148
Williams (2000) 24mths *NHS – IBD*	951	1046	95	+47.50
Williams (2000) 24mths ***patients-IBD***	122	115	7	−3.50
Robinson (2001) 14mths ***patients-IBD***	8.92	0.86	8.06*	−6.91

**NB:** Summary of mean total NHS financial cost per patient (in time specified) and mean total financial cost to patient (in time specified).

* P<0.001.

Average difference in **NHS cost** per patient per year is £89.06 range £+47.50 to −150.75 in favour of the intervention.

Average difference in **Patient cost** per year is £5.21 range £3.50–6.91 in favour of the intervention.

**NB.** Koinberg (2009) costs originally published in Euros, converted to Sterling using Jan 2006 exchange rates (0.688 Euros to the pound) (http://www.x-rates.com/cgi-bin/hlookup.cgi).

There was no perceived difference between measured costs in the intervention and control groups at 12 months, in the study of people with BC [12].

Kennedy and colleagues [15] reported reduced costs in the intervention group for all outcomes at 12 months in comparison to the control group. These results included reduced costs from GP visits, IBD outpatient visits, IBD inpatient stays and medication. The results from Williams and colleagues' [17] study were more mixed. The findings reported significantly reduced mean costs for investigations made in secondary care (m = £198 vs m = £257; p = 0.032) and for total cost to secondary care (£582 vs £611, p = 0.012) at 24 months. However, the total drug, primary care, NHS and societal costs per patient were all reported to be marginally higher in the intervention group than in the control group at 24 months; these differences are not significant.

One study on RA reported about costs [19] and found that the PIC intervention significantly reduced costs to the NHS over one year (£208 per patient per year) in comparison to usual follow up (£313 per patient per year, p<0.001).

Robinson and colleagues [16] reported a reduced cost in terms of patient's time (including travelling to and from appointments) at 14 months (PIC m = 1 hr ±2.95 vs m = 6.2 hr ±7.1; p<0.0001). Robinson and colleagues' [16] study also reported a significant difference in mean cost for patients between the groups at 14 months (m = £0.86±3.41 vs 8.92±18.30 respectively; p<0.0001). Williams and colleagues [17] also found cost savings for patients in the intervention group (see Table 1).

### Exploration of relationships

Figure 5 portrays one theory about how the PIC system could lead to a more effective and satisfactory service without harming patients. Additional boxes (with an interrupted border) are where the studies included in this review may inform the future implementation of the PIC system. Establishing definitive links between the characteristics of the intervention and the reported outcomes was not possible from the small amount of variable literature that was available for inclusion in this review. However, below are some observations regarding possible links which should be considered in any further research in this area.

Four studies [12], [13], [15], [16] described a similar PIC system with each involving written information to support the patient, a telephone helpline, an annual check-up and an initial consultation (Brown and colleagues report no initial consultation). From the results across outcomes it could be argued that those studies tended to produce better results for the PIC system. However, the Hewlett and Kirwan [19]–[21] studies also reported positive results for the PIC system without the use of written information or an initial consultation. The methods suggest that a large amount of verbal information was supplied by the consultant to individuals in the intervention group in these studies. Interestingly, there was some suggestion that studies in which written information was provided in addition to a telephone helpline and an annual check-up were associated with greater long-term cost savings to the NHS and the patient.

Another feature of some of the PIC interventions was that much of the care responsibility was handed back to the primary care physician [17], [19]–[22], though this was primarily the case in those that did not have any written information for the patient. This would have meant that the patient could contact their primary care physician with any concerns about their condition in the first instance. The primary care physician was then guaranteed speedy access to a specialist consultant when needed or the patient was allowed to contact the hospital directly. However, it is possible that whilst support from the primary care physician may replace written information adequately in terms of clinical outcomes, the impact on resources and costs was less clear. In some instances, the number of visits to the GP and the cost of the intervention to primary care could have increased whilst appointments and costs in secondary care decreased.

## Discussion

We identified eight studies of the impact of a PIC system on the clinical outcomes of patients with BC, IBD and RA in secondary care. Although there were limitations in the quality of reporting, which indicates that a number of the studies may have been affected by bias, and the level of heterogeneity in populations and outcomes were considerable, there was some evidence that the PIC system of appointment scheduling was not harmful to patients with BC, IBD and RA, and may be associated with savings in health service resources, costs and clinician time. In the included studies there were minimal differences in clinical outcomes between traditional appointment scheduling and the PIC system, suggesting that PIC follow up was as good as traditional models at delivering care. The differences observed could be due to the nature of the disease areas, i.e. the relapsing and remitting nature of IBD and RA, compared to the “remission or recurrence” in malignancies like BC (e.g. people can learn to manage their own symptoms with appropriate guidance better in the former than the latter). It might not be expected that a change in service would necessarily lead to an improvement in the way symptoms are experienced by the patient but no further data was available to inform this observation. However, it is important to note if symptoms become significantly worse. There was evidence that the PIC system could be associated with a reduced number of booked appointments which allows clinician and patient time to be used more efficiently. This would allow for more autonomy within the service to treat those who need immediate care quickly. Several studies reported financial cost savings to the NHS and to patients, however, whether the PIC system is cost effective in the long term is unclear and may be dependent on the condition being considered.

Though the results of this review imply benefits in the use of the PIC system without harming patients they must be taken in the context of the various limitations and potential biases within the included studies. The most obvious harm risk in terms of clinical outcomes is believed to result from situations where the patient fails to request an appointment at the time of relapse or escalation of their condition and therefore symptoms become worse and possibly critical. This may increasingly be the case where PIC interventions do not include a ‘safety-net’ appointment system or where clinicians are unable to select ‘appropriate’ patients for the PIC pathway. A less apparent harm risk may occur if during a “routine” traditional appointment there are elements of preventative healthcare or patient education that occur, which are not covered during an “urgent” PIC appointment. This risk can probably be circumvented by incorporating an appropriate check list into a safety net appointment. There are no statistically significant harms reported by any study in terms of clinical outcomes, however, some of the studies (n = 4) excluded participants who had not completed all the questionnaires or diaries from the final analyses. From these studies it is clear that approximately 18 patients withdrew from the studies due to recurrence of illness, 34 refused to complete the final questionnaires or were otherwise lost to follow-up and in one study approximately 19 patients died during participation [21] so the potential for harms to have been recorded may have limited. In addition to the bias within the studies, publication bias may also have influenced the results of the review, reflecting the bias towards pursuing ideas and publishing studies with positive outcomes.

All of the included studies were conducted within the UK. There are a number of possible explanations for this: 1) other terms for this approach may be used in different health care systems; 2) PICs are implemented in secondary care without evaluation of their impact and hence no primary research is available; 3) evaluation of PICs may be hidden within health departments with limited access to and awareness of the final report documents. We are confident that our search terms are likely to have identified all appropriate studies published in English. Our search strategy did not include grey literature (i.e. literature informally published, such as technical reports from government agencies or scientific research groups, working papers from research groups or committees, white papers, and preprints that may be difficult to trace via published journals and monographs because it is not widely accessible) from outside the UK and this is a limitation, although it seems unlikely that grey literature would contain randomised evaluations of PICs. Contact with professionals identified an ongoing trial investigating the use of outpatient on-demand clinics for people with Chronic Obstructive Pulmonary Disease in the Netherlands (http://clinicaltrials.gov/ct2/show/NCT00556816).

It is perhaps surprising that, despite the promising nature of the PIC system and the direction of national guidelines for RA and IBD, such limited research has been conducted to establish the barriers to or even the key components of implementing this system of care. Recent guidelines for the management of RA [23], [24] and IBD [25] in adults have all suggested that patients with these conditions should be able to access a specialist as soon as possible if the state of their condition worsens. Recent qualitative research in people with IBD has also reported that patients want flexibility and choice, they do not want to be discharged from secondary care and desire the specialist to still be involved in overall management [26]. The PIC approach to outpatient care is clearly in line with these guidelines and would also fall in line with guidelines for other chronic diseases. For example, the UK Department of Health National Service Framework for long term [4] conditions states, in relation to care for people with Parkinson's Disease, that there should be enough flexibility to allow for both planned reviews and unplanned reviews when a person's condition suddenly deteriorates or their circumstances change [27]. Furthermore, the British Thoracic Society guidelines also discuss outpatient clinic management in this way and a recent study has used similar processes of minimising clinic time for well patients and giving them fast access to clinician care should they deteriorate [28].

If it is true, as suggested by our review, that patients are not likely to come to harm as a result of follow-up initiated by themselves, then there may be substantial gains in efficiency for health care systems to adopt this approach in suitable conditions and with appropriately selected patients. Giving patients the option of either form of follow-up may be important in avoiding harm and ensuring the most appropriate patients use the service. Importantly, five out of eight identified studies retained an annual or biennial consultation as a safety-net in the intervention groups. There is a clear need for long term randomised controlled trials of this type of appointment system using comparable outcome data. Quantitative and qualitative evaluations of PIC implementations to enable a better understanding of clinical and efficiency outcomes and investigation of context-dependent influences including the professional and organisational barriers to implementing PICs should also be conducted, as suggested by Whear and colleagues [29]. Patient initiated clinic systems should include some form of ‘safety-net’ and allow for how clinicians and patients would like to use the system e.g. allowing patient choice and/or clinician selection into specific care pathways.

## Conclusions

The UK policy context is ripe for evidence-based, patient-centred services to be implemented and evaluated, especially where health professionals' and patients' wasted time and costs can be minimised without reducing the quality of health care or patient outcomes. Present research on the PIC system has presented some positive findings but implementation should remain cautious with ongoing evaluation of its long term outcomes and costs.

## Supporting Information

Table S1 **Data Extraction and Quality Appraisal form.**
(DOCX)

Table S2 **Characteristics of patient initiated clinics in included studies.**
(DOCX)

Table S3 **Outcome data from original articles.**
(DOCX)

Figure S1 **Key for** **Figure 3** **.**
(TIF)

Checklist S1 **Prisma checklist.**
(PDF)

## References

[1] DoH (2001) The Expert Patient: A New Approach to Chronic Disease Management for the 21st Century. London: Department of Health. 35.

[2] Hehir M, Hewlett S, Mitchell K, Kirwan J, Memel D, et al (2001) What happens in RA outpatient clinics? Rheumatology 40: s146.

[3] HES (2010) Outpatient data: SHA and provider level analysis 2008–09 and 2009–10. ONS: NHS Information Centre for Health and Social Care. http://www.hscic.gov.uk/searchcatalogue?productid=2805&q=%2822hospitaloutpatientactivity%2822&topics=2800%2802fHospitalcare&sort=Relevance&size=2810&page=2801#top.

[4] DoH (2004) Choose & Book – Patient's Choice of Hospital and Booked Appointment. London: Department of Health. p. 16.

[5] WHO (2002) Innovative Care for Chronic Conditions: Building Blocks for Action. Geneva: World Health Organisation. 103 p.

[6] Rose KD, Ross JS, Horwitz LI (2011) Advanced access scheduling outcomes a systematic review. Archives of internal medicine 171: 1150–1159. 21518935 10.1001/archinternmed.2011.168 PMC3154021

[7] Robinson L, Chen R (2010) A comparison of traditional and open-access policies for appointment scheduling. Manufacturing and Service Operations Management 12: 330–346.

[8] Liu N, Ziya S, Kulkarni VG (2010) Dynamic scheduling of outpatient appointments under patient no-shows and cancellations. M&Som-Manufacturing & Service Operations Management 12: 347–364.

[9] Nuffield (2011) Health and Social Care Bill: Second reading, House of Lords. London: Nuffield Trust. 16 p.

[10] CRD (2009) Systematic Reviews: CRD's guidance for undertaking reviews in healthcare. York: Centre for Reviews and Dissemination. 294 p.

[11] Popay J, Roberts H, Sowden A, Petticrew M, Arai L, et al.. (2006) Guidance on the conduct of systematic reviews. London: Economic and Social Research Council.

[12] Brown L, Payne S, Royle G (2002) Patient-initiated follow-up of breast cancer. Psycho-Oncology 11: 346–355. 12203747 10.1002/pon.576

[13] Koinberg I, Engholm G, Genell A, Holmberg L (2009) A heath economic evaluation of follow-up after breast cancer surgery. Acta Oncologica 48: 99–104. 18766474 10.1080/02841860802314712

[14] Sands A, Adams N (2009) A comparison of patient-initiated versus conventional follow-up for rheumatoid arthritis. Journal of Pain Management 1: 391–400.

[15] Kennedy A, Nelson E, Reeves D, Richardson G, Roberts C, et al.. (2003) A randomised controlled trial to assess the impact of a package comprising a patient-orientated, evidence-based self-help guidebook and patient-centered consultations on disease management and satisfaction in inflammatory bowel disease Health Technology Assessment 7. 10.3310/hta7280 14567905

[16] Robinson A, Thompson DG, Wilkin D, Roberts C, Grp NWGR (2001) Guided self-management and patient-directed follow-up of ulcerative colitis: a randomised trial. Lancet 358: 976–981. 11583752 10.1016/S0140-6736(01)06105-0

[17] Williams JG, Cheung WY, Russell IT, Cohen DR, Longo M, et al (2000) Open access follow up for inflammatory bowel disease: Pragmatic randomised trial and cost effectiveness study. British Medical Journal 320 (7234): 544–548. 10.1136/bmj.320.7234.544 PMC27297 10688560

[18] Chattopadhyay C, Hickey PM (2008) Are routine appointments cost-effective in modern rheumatology practice? A comparative outcome audit of routine follow-ups and patient initiated consultations. Arthritis and Rheumatism 58: S885–S885.

[19] Hewlett S, Mitchell K, Haynes J, Paine T, Korendowych E, et al.. (2000) Patient-initiated hospital follow-up for rheumatoid arthritis. SO: Rheumatology (Oxford, England): 990–997. 10.1093/rheumatology/39.9.990 10986304

[20] Hewlett S, Kirwan J, Pollock J, Mitchell K, Hehir M, et al (2005) Patient initiated outpatient follow up in rheumatoid arthritis: six year randomised controlled trial. BMJ 330: 171. 15546895 10.1136/bmj.38265.493773.8F PMC544988

[21] Kirwan JR, Mitchell K, Hewlett S, Hehir M, Pollock J, et al (2003) Clinical and psychological outcome from a randomized controlled trial of patient-initiated direct-access hospital follow-up for rheumatoid arthritis extended to 4 years. Rheumatology 42 (3): 422–426. 10.1093/rheumatology/keg130 12626791

[22] Sheppard C, Higgins B, Wise M, Yiangou C, Dubois D, et al (2009) Breast cancer follow-up: a randomised controlled trial comparing point of need access versus routine 6-monthly clinical review. European Journal of Oncology Nursing 13: 2–8. 19119079 10.1016/j.ejon.2008.11.005

[23] NICE (2009) Rheumatoid arthritis: The management of rheumatoid arthritis in adults. London: National Institute for Health and Clinical Excellence. 35 p.

[24] Luqmani R, Hennell S, Estrach C, Basher D, Birrell F, et al (2009) British Society for Rheumatology and British Health Professionals in Rheumatology guideline for the management of rheumatoid arthritis (after the first 2 years). Rheumatology 48: 436–439. 19174570 10.1093/rheumatology/ken450a

[25] Mowat C, Cole A, Windsor A, Ahmad T, Arnott I, et al (2011) Guidelines for the management of inflammatory bowel disease in adults. Gut 60: 571–607. 21464096 10.1136/gut.2010.224154

[26] Kemp K, Griffiths J, Campbell S, Lovell K (2013) A qualitative exploration of the follow up needs of patients with inflammatory bowel disease. Journal of Crohn's and Colitis 7: S246–S247. 10.1016/j.crohns.2013.03.001 23541150

[27] DoH (2005) The National Service Framework for long term conditions. London: Crown Copyright. 106.

[28] Turner AM, Dalay SK, Talwar A, Snelson C, Mukherjee R (2013) Reforming respiratory outpatient services: a before-and-after observational study assessing the impact of a quality improvement project applying British Thoracic Society criteria to the discharge of patients to primary care. Primary Care Respiratory Journal 22: 72–78. 10.4104/pcrj.2013.00013 PMC6442754 23443226

[29] Whear R, Abdul-Rahman A, Thompson-Coon J, Boddy K, Perry M, et al. Patient initiated clinics for patients with chronic or recurrent conditions managed in secondary care: a systematic review of patient reported outcomes and patient and clinician satisfaction. BMC Health Services Research (under review): personal communication. 10.1186/1472-6963-13-501 PMC3879028 24289832

