# Supplementary material for: The Clinical Effectiveness of Patient Initiated Clinics for Patients with Chronic or Recurrent Conditions Managed in Secondary Care: A Systematic Review
Source: PLoS One. 2013 Oct 7;8(10):e74774. doi: 10.1371/journal.pone.0074774 (PMC3792120; doi:10.1371/journal.pone.0074774)
Supplement: Table S1 — Data Extraction and Quality Appraisal form. (DOCX) [file pone.0074774.s001.docx]

**Table S1 - Data Extraction and Quality Appraisal form**

| Bibliographic details  Reference no:  First author:  Title:  Year:  Citation: | |
| --- | --- |
| STUDY  Country:  Setting:  Recruitment dates:  Study design:  Funding source:  Notes: | INTERVENTION  Description of Intervention:  Description of Comparator:  Notes:  Is the intervention described in sufficient detail for it to be replicated elsewhere? |
| SUBJECTS  Total number:  Inclusion criteria:  Exclusion criteria: |  |
| Patient characteristics   \| \| Mean (SD) \| **control** \| \| **intervention** \| \| \| --- \| --- \| --- \| --- \| --- \| \| N: \|  \| \|  \| \| \| Age *yrs*: \|  \|  \|  \|  \| \| Sex: \|  \|  \|  \|  \| \| M \|  \|  \|  \|  \| \| F \|  \|  \|  \|  \| \|  \|  \|  \|  \|  \| \|  \|  \|  \|  \|  \| \|  \|  \|  \|  \|  \| \| \| --- \| --- \| --- \| --- \| --- \| --- \| --- \| --- \| --- \| --- \| --- \| --- \| --- \| --- \| --- \| --- \| --- \| --- \| --- \| --- \| --- \| --- \| --- \| --- \| --- \| --- \| --- \| --- \| --- \| --- \| --- \| --- \| --- \| --- \| --- \| --- \| --- \| --- \| --- \| --- \| --- \| --- \| --- \| --- \| --- \| --- \| | OUTCOME MEASURES  Primary outcome measure:  Secondary measures:  Method of assessing outcomes:  Length of follow-up:  Notes:  This section should include a list of outcomes measured and the methods of measuring them not the results.  Are the questionnaires validated?  Is the primary outcome measure specified? |

| Results  Total number of participants enrolled:  Number lost to follow-up (if appropriate):   \| \|  \| **control** \| \| **intervention** \| \| \| --- \| --- \| --- \| --- \| --- \| \|  \| n \| mean (sd) \| n \| mean (sd) \| \| *Outcomes* \|  \|  \|  \|  \| \|  \|  \|  \|  \|  \| \|  \|  \|  \|  \|  \| \|  \|  \|  \|  \|  \| \|  \|  \|  \|  \|  \| \|  \|  \|  \|  \|  \| \|  \|  \|  \|  \|  \| \| \| --- \| --- \| --- \| --- \| --- \| --- \| --- \| --- \| --- \| --- \| --- \| --- \| --- \| --- \| --- \| --- \| --- \| --- \| --- \| --- \| --- \| --- \| --- \| --- \| --- \| --- \| --- \| --- \| --- \| --- \| --- \| --- \| --- \| --- \| --- \| --- \| --- \| --- \| --- \| --- \| --- \| --- \| --- \| --- \| --- \| --- \|   Adverse events:  Type of analysis (intention to treat, per protocol):  Results of study analysis (e.g. Dichotomous: odds ratio, risk ratio and confidence intervals, p-value; Continuous: mean difference, confidence intervals):  Notes: |
| --- | --- | --- | --- | --- | --- | --- | --- | --- | --- | --- | --- | --- | --- | --- | --- | --- | --- | --- | --- | --- | --- | --- | --- | --- | --- | --- | --- | --- | --- | --- | --- | --- | --- | --- | --- | --- | --- | --- | --- | --- | --- | --- | --- | --- | --- | --- |
| quality appraisal   \| 1. Study design \| {RCT; X-over trial: CCT; pre-post study} \| \| --- \| --- \| \| 2. Were the study eligibility criteria specified? \| {yes; no; partial} \| \| 3. Was a power calculation performed? \| {yes; no} \| \| 4. Is the sample size adequate? \| {yes; no; unclear} \| \| 5. Is the number randomized stated? \| {yes; no; not applicable} \| \| 6. Is the study properly randomized?┼ \| {yes: no; not applicable; unclear} \| \| 7. Is allocation of treatment concealed? ╪ \| {yes; no; not applicable; unclear} \| \| 8. Are adequate baseline details described? \| {yes; no; partial} \| \| 9. Are groups similar at baseline? \| {yes; no; partial; not applicable} \| \| 10. Are baseline imbalances adequately adjusted for in the analysis? \| {yes; no; not applicable; unclear} \| \| 11. Are similar co-interventions administered? \| {yes; no; unclear; not applicable} \| \| 12. Are patient’s blinded to treatment allocation? \|  \| \| 13. Are outcome assessors blinded? \| {yes; no; unclear} \| \| 14. Is compliance with treatment adequate? \| {yes; no; unclear; not reported} \| \| 15. Were any sub-group analysis justified? \| {yes; no; not applicable} \| \| 16. Were data collection tools shown or known to be valid  for the outcome of interest?± \| {yes; no; unclear} \| \| 17. Were the data collection tools known or were shown to be  consistent and accurate in measuring the outcome of interest?* \| {yes; no; unclear} \| \| 18. Were all study participants accounted for? \| {yes; no} \| \| 19. Are data analyses appropriate? \| {yes; no; partial; unclear} \| \| 20. Is analysis conducted on an ITT basis? \| {yes; no; not applicable} \| \| 21. Are greater than 80% of patients included in the follow-up assessment? \| {yes; no; unclear} \| \| 22. Are the conclusions supported by the results? \| {comment} \| \| 23. Generalisability \| {comment} \| \| 24. Inter-centre variability \| {comment} \| \| 25. General comments \| {comment} \|   ┼ Adequate approaches top sequence generation: computer-generated random numbers, random number tables; inadequate approaches: use of alternation, case record numbers, birth dates or week days  ╪ Adequate approaches to concealment allocation: centrally or pharmacy-controlled randomisation, inadequate approaches: serially numbered envelopes, use of alternation, open random number lists  ± The tools are known to be valid or were shown to measure what they are intended to measure  * The tools are known to be reliable or were shown to be consistent and accurate in measuring the outcome of interest (e.g. test-retest, Cronbach’s alpha, interrater reliability) |
| GENERAL COMMENTS  Data extraction performed by: Date:  Data extraction checked by: Date: |
