# Supplementary material for: The Clinical Effectiveness of Patient Initiated Clinics for Patients with Chronic or Recurrent Conditions Managed in Secondary Care: A Systematic Review
Source: PLoS One. 2013 Oct 7;8(10):e74774. doi: 10.1371/journal.pone.0074774 (PMC3792120; doi:10.1371/journal.pone.0074774)
Supplement: Table S2 — Characteristics of patient initiated clinics in included studies. (DOCX) [file pone.0074774.s002.docx]

**Table S2 – Characteristics of patient initiated clinics in included studies**

| **Study** | **Written information** | **Telephone help line** | **Initial consultation** | **Annual checkup** |
| --- | --- | --- | --- | --- |
| **Brown (2002)** | √ | √ | × | √ |
| **Koinberg (2009)** | √ | √ | √ | √ |
| **Sheppard (2009)** | × | √ (+ GP informed) | × | √ |
| **Kennedy (2003)** | √ (guidebook) | √ | √ | √ (some cases) |
| **Robinson (2001)** | √ (guidebook) | √ | √ | √ (some cases) |
| **Williams (2000)** | × | √ (+ GP contact) | × | √ (24mths) |
| **Chattopadyay (2008)** | Not described | | | |
| **Hewlett (2000)** | × | √ (+ GP contact) | × | × |
| **Kirwan (2003)** | × | √ (+ GP contact) | × | √ (24mths) |
| **Hewlett (2005)** | × | √ (+ GP contact) | × | √ (24mths) |
