# Supplementary material for: The Clinical Effectiveness of Patient Initiated Clinics for Patients with Chronic or Recurrent Conditions Managed in Secondary Care: A Systematic Review
Source: PLoS One. 2013 Oct 7;8(10):e74774. doi: 10.1371/journal.pone.0074774 (PMC3792120; doi:10.1371/journal.pone.0074774)
Supplement: Table S3 — Outcome data from original articles. (DOCX) [file pone.0074774.s003.docx]

**Table S3 - Outcome data from original articles**

| **Study** | **Outcomes** | **Results** | | |
| --- | --- | --- | --- | --- |
|  |  | **Intervention**  **M (SD)** | **Standard Care**  **M (SD)** | **Comments** |
| Sheppard et al, 2009 (BC) | No. Of relapses in last year | 5 (4%) | 4 (4%) | N (%) |
| Kennedy et al, 2003 (IBD) | No. Of relapses in last year | 1.8 (2.2) | 2.2 (2.5) | P=0.013 |
|  | No. Of medically defined relapses (based on diaries) | 1.2 (1.5) | 1.2 (1.5) | P=0.90 |
|  | Relapse duration (days) | 25.8 (47.9) | 18.4 (36.0) | P=0.19 |
| Robinson, 2001  (IBD) | *At 14 mths*  At least one relapse | 61% (m=1.53) | 49% (m=1.93) | P<0.0001 |
|  | Relapse with treatment in 2 days | 97% | 63% |  |
|  | Relapse self-treated | 112 (96%) | 30 (50%) | P<0.0001 |
|  | Inappropriate self treatment | 6 (5%) | 30 (30%) |  |
| Chattopadyay, 2008  (RA) | Rate of all interventions | 96.7% | 52.2% | P<0.00001 |
|  | Rate of meaningful interventions | 66.7% | 30.1% | P<0.0001 |
| Hewlett, 2000  (RA) | *At 24 months*  Pain | 3.9cm | 4.8cm | P<0.05 |
|  | Change in pain | +0.4cm | +1.6cm | P<0.01 |
|  | Change in disease activity | -8% | +17% | NS |
|  | Mean disability | Improved 8% | Worsened 4% | NS |
|  | Larsen Index | 48.4 (20.4) | 50.1 (27.3) | NS |
| Kirwan, 2003  (RA) | *Change in scores 0-48mths*  Disability (HAQ) | 0.21 | 0.25 |  |
|  | Pain | 1.3 | 1.6 |  |
|  | Disease activity | 0.3 | 0.2 |  |
|  | Early am stiffness | 54 | 4 |  |
|  | C reactive protein | 0 | -2 |  |
|  | Plasma Viscosity | 0.02 | -0.04 |  |
|  | Haemoglobin | 0.1 | 0.1 |  |
|  | Larsen Index: |  |  |  |
|  | - Right elbow | -13 | -18 |  |
|  | - Left elbow | -14 | -24 |  |
|  | - Right knee | -10 | -13 | P<0.05 |
|  | - Left knee | -13 | -16 |  |
|  | Grip strength R | -2.1 | -0.9 |  |
|  | Grip strength L | -3.1 | -0.8 |  |
| Hewlett, 2005  (RA) | *Median change and range at 6 years*  Pain | 1.25 (-0.40 - 3.25) | - 1. (-1.00 – 3.60) | P=  0.91 |
|  | Disease activity | 0.25 (-1.35 - 2.80) | 0.25 (-0.88 – 2.80) | 0.49 |
|  | Early morning stiffness | 0 (-10.0 - 33.0) | 5.0 (-15.0 – 20.0) | 0.80 |
|  | C Reactive protein | -0.95 (-12.0 - 20.5) | -3.00 (-8.4 – 3.0) | 0.62 |
|  | Plasma viscosity | 0.07 (-0.01 - 0.14) | 0.07 (-0.04 – 0.16) | 0.78 |
|  | Haemoglobin | 0 (-6 - 9) | 2 (-5.5 – 7.8) | 0.39 |
|  | Disability | 0.19 (-0.125 - 0.75) | 0.25 (0 - 0.75) | 0.39 |
|  | Grip strength R | -4.0 (-10 - 0) | -2.0 (-5.5 – 2.0) | 0.13 |
|  | Grip strength L | -4.0 (-10 - 0) | -1.0 (-7.0 – 0) | 0.07 |
|  | Larsen Index: |  |  |  |
|  | - Right elbow | 17.0 (-35.0 – 0) | -25.5 (-40.5 – 14.5) | 0.04 |
|  | - Left elbow | 15.0 (-25.0 – 0) | -24.5 (40.3 - -4.0) | 0.02 |
|  | - Right knee | -4.0 (-20.0 – 7.0) | -8.0 (-30.0 – 7.0) | 0.60 |
|  | - Left knee | -5.0 (-20.0 – 5.0) | -8.5 (-26.3 - -8.5) | 0.65 |
|  | - Both hands | 14 (0 - 27) | 9 (2 - 23) | 0.69 |
