# Supplementary figures and images for: The Clinical Effectiveness of Patient Initiated Clinics for Patients with Chronic or Recurrent Conditions Managed in Secondary Care: A Systematic Review
Source: PLoS One. 2013 Oct 7;8(10):e74774. doi: 10.1371/journal.pone.0074774 (PMC3792120; doi:10.1371/journal.pone.0074774)

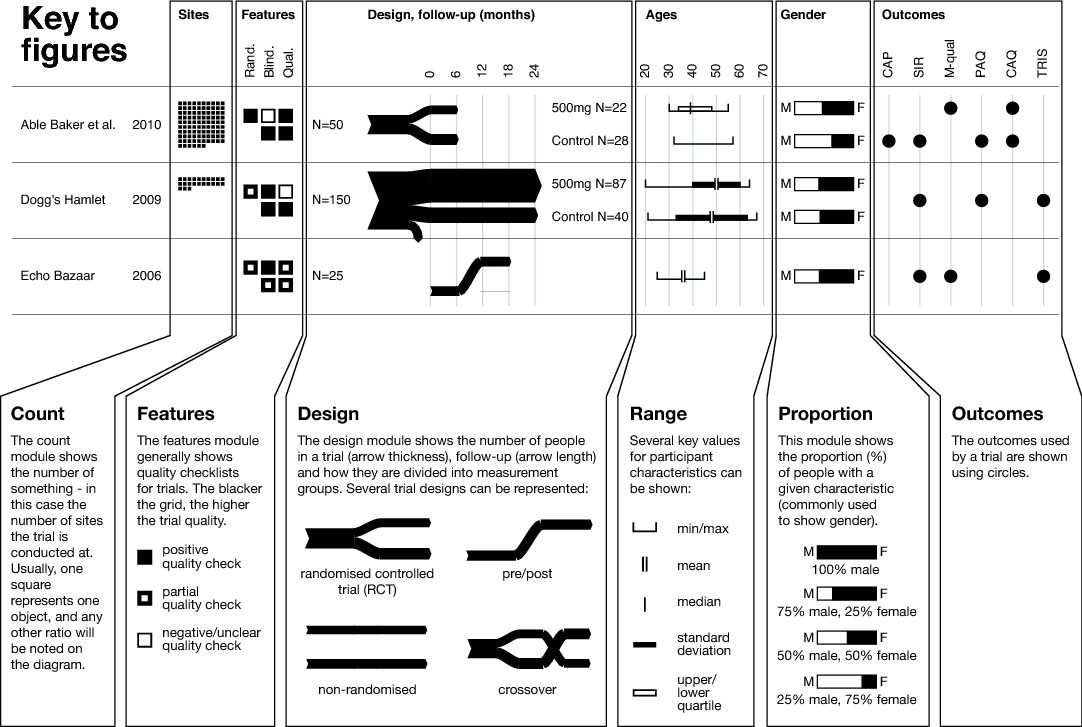

Supplement: Figure S1 — Key for Figure 3 . (TIF) [file pone.0074774.s004.tif]
